# The biomechanical study of rupture of Achilles Tendon and repair by different suture techniques

**DOI:** 10.12669/pjms.343.14842

**Published:** 2018

**Authors:** Chang-Chun Yang, Xiao Yu, Zong-hui Guo, You-wei Fu

**Affiliations:** 1 Dr. Chang-Chun Yang, MD Department of Orthopedics, Ningbo No.2 Hospital, Ningbo, 315010, China; 2 Dr. Xiao Yu, PhD. Department of Orthopedics, Ningbo No.2 Hospital, Ningbo, 315010, China; 3 Dr. Zong-hui Guo, MD Department of Orthopedics, Ningbo No.2 Hospital, Ningbo, 315010, China; 4 Dr. You-Wei Fu, MD Department of Orthopedics, Ningbo No.2 Hospital, Ningbo, 315010, China

**Keywords:** Achilles tendon repair, Anchor suture, Biomechanical study, Rupture of Achilles tendon

## Abstract

**Objective:**

To study the biomechanical properties of different suture methods, and to provide evidence for the clinical application of this suture methods in repairing acute Achilles tendon rupture.

**Methods:**

Twenty four fresh frozen cadaver Achilles tendon specimens were collected and randomly divided into three groups (n=8), Group-A Bunnell suture method, Group-B Bosworth suture and Group-C anchor suture respectively. 5 N tensions were applied to tighten the tendon. The actual length of the tendon between the upper and lower clips was measured with a ruler. The length of the long axis and the short axis of the three sections of the tendon was measured by vernier caliper. The cross sectional area of the tendon was calculated according to the elliptical area formula and the mean value was obtained.

**Results:**

There was no significant difference in the length and cross-sectional area of each tendon among three groups (F=0.26, *P*=0.86; F=0.09, *P*=0.96). There was no significant difference in the maximum load of tendon and failure displacement in Group A and B (*P*>0.05). The maximal load of Group-C was significantly larger than that of Group A and B (*P*<0.05), and there was no significant difference between the failure displacement and Group A and B (*P*>0.05).

**Conclusion:**

Three suture methods can provide good biomechanical properties, but the anchor suture is more effective in solving the shortcomings of traditional methods. It is a safe and effective method, and is worthy of promotion.

## INTRODUCTION

The Achilles tendon rupture is a serious injury and its incidence is 6-37 per one hundred thousand.^1^ The rupture point of tendon is often located 2~6cm above the tendon attachment point. About 75% of the tendon injuries are related to sports. A lot of surgical methods have been developed with the deep understanding of Achilles tendon rupture, such as V-Y shortening, reinforce by sural tendon flap, plantar tendon, peroneus brevis tendon, flexor digitorum longus tendon, fascia strip or combined tendon flap of gastrocnemius muscle. There are different reinforces by using the DuPont polyester sheet, carbon fiber strip, proteoglycan line or polyethylene net. A number of suture methods have also been developed, like Kirschmayer, Motta, Kakiuchi, Bunnel, Bosworth, Lindholm and V-Y tendon plasty. Different surgical treatments correspond to different injuries.^2^ However; there is still a lot of debate about the choice of various suture methods for the rupture of the Achilles tendon in the current course of diagnosis and treatment. If the suture method is chosen improperly, it will affect the local biomechanical environment of the foot and a series of complications such as rupture of the Achilles tendon, Achilles tendon contracture and limited motion of the ankle will cause great pain to the patient. Therefore, we believe that it is necessary to study the biomechanics of Achilles tendon injury and different suture methods. In this study, a proper method of Achilles tendon suture after Achilles tendon injury is discussed from a biomechanical point of view.

## METHODS

Twenty four fresh specimens of Achilles tendon with its insertion of the calcaneal tuberosity were chosen for this study (Provided by Ningbo University, School of medicine). All the specimens have the normal shape without defect or injury. By X-ray examination, the diseased of degeneration, rupture and structural malformation in the calcaneal tuberosity were ruled out.

### Experimental equipment

2T torsion load testing machine (Changchun Mechanical Science Research Institute Co., Ltd.), TwinFIX TI 3.5 Suture Anchor (Smith & nephew Medical Co., Ltd, USA), ETHIBOND EXCEL Polybutylate coated braided Polyester Suture (Johnson Medical Co., Ltd, USA).

### Experimental methods Specimen preparation

The specimens were exposed at the room temperature for 12 hours before experiment. The Achilles tendon should be kept moist to ensure its tension. The Achilles tendon with its insertion of the calcaneal tuberosity was kept intact and the other part of the calcaneal tuberosity was fixed in the specimen fixing box and fixed on the base of the torsion load testing machine. The other end of the Achilles tendon should be fixed in the upper fixing device of the testing machine and the tension load could be adjusted as 0N.Then all the Achilles tendons in the specimens were cut by the bistoury to simulate the rupture of the Achilles tendon.

### Modeling

Twenty four fresh frozen cadaver Achilles tendon specimens were collected and randomly divided into 3 groups. Each group has eight specimens.

1. ***Groups-A is Bunnell Suture method:*** The Kirschner needle was used to guide the calcaneus tubercle. A certain point of the wire ends distal from the calcaneal from the foot part penetrates through with a button fixed on the wire can be placed out of the skin.
2. ***Groups-B is Bosworth Suture method:*** The tendon is sewn into a tube, and the smooth surface remains outside the tendon and then turns downward. After the suture was strengthened at the proximal end, the defect was traversed down to the distal end. At the distal end, the tendon was tightened and the suture was tightened. The remaining tendons were turned upwards and sutured to the posterior side of the proximal end. Then the spare plantar tendon is fanned out to form a smooth fan patch around 3cm, covering the Achilles tendon repair. The edges are fixed with a number of needles to prevent movement.
3. ***Group-C is anchor suture method:*** 1~2 thread anchors were screwed at the insertion of calcaneal Achilles tendon. The angle between the anchorage and the Achilles tendon is 45°, and the ankle joint is in neutral position, hence the 2 ultra strong sutures at the tail of the anchorage were used to weave both sides of the Achilles tendon with Kessler or Krackow. The length of the braided suture of the Achilles tendon is 3 cm with TwinFIX TI 3.5 Suture Anchor to strengthen the suture of the reduplicated Achilles tendon.

### Biomechanical test

**Fig.1:**
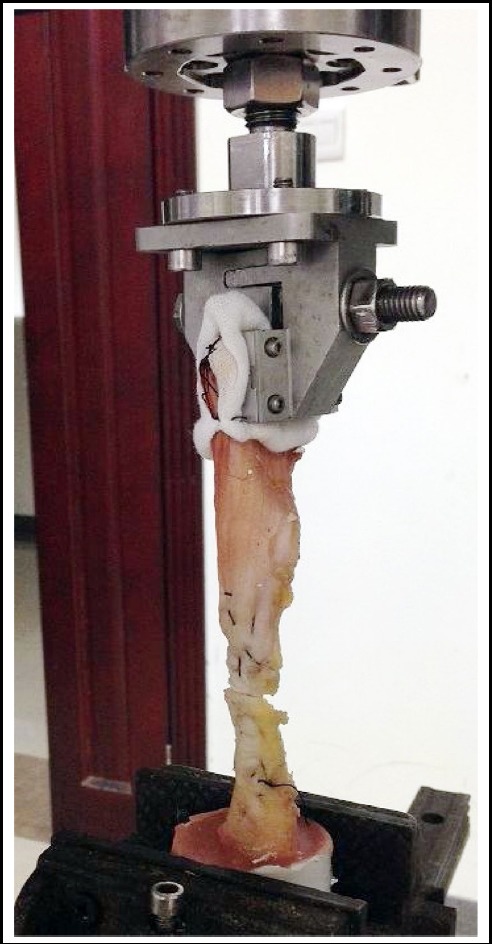
The biomechanical test of rupture of Achilles tendon and repair by different suture techniques.

The specimens were mounted on the 2T torsion load testing machine, and the specimens were grouped and marked. The test machine is set up to the computer in front of the computer, and the machine is set to two attenuation multiplier, the sensor range is 50N, the loading speed is 15mm/min, and then the test machine and computer are started simultaneously. Keep the Achilles open and repeat the above methods (Fig.1).

### Inspection record item

Length and cross section of cadaver Achilles tendon, The length and cross section diameter of the specimen is measured by a ruler and a vernier caliper. The shape of the cross section is flat; D1 and D2 are two dimensions in vertical direction respectively. Finally, the average cross section area is calculated. With the speed of 15mm/min stretching, the formation of computer automatic recording 2mm clearance and 100N tensile load displacement, maximum rupture load (peak load), tensile and pull in rupture.

### Statistical analysis

SPSS 23.0, statistical software was used for analysis. The data were expressed with mean ± standard deviation, and the analysis of variance was compared between groups. Multiple comparisons using SNK test. The test level is of α=0.05.

## RESULTS

The actual lengths of tendons in Group A, B and C were (128.88±0.56), (128.53±0.45) and (128.65±0.51) mm respectively, and the cross-sectional area was (141.95±2.56), (139.89±3.52), and (138.92±2.98) mm^2^, respectively. There was no significant difference in the length and cross section of the tendon in each group (F=0.26, P=0.86; F=0.09, P=0.96).

### Group A and B

No statistical analysis of the specimens was excluded because of slipping; failure due to suture rupture.

### Group-C

No statistical analysis of the specimens was excluded because of slipping; sutures slipping from tendon tissue together.

There was no significant difference in the maximum load of tendon and failure displacement in Group A and B (P>0.05). The maximal load of Group-C was significantly larger than that of Group A and B (P<0.05), and there was no significant difference between the failure displacement in Group A and B (P>0.05). Table-I.

**Table-I:** Comparison of the biomechanical test results in each group (n=8, X±S).

Group	Maximum load (N)	Failure displacement (mm)
A	111.36±4.94	20.25±1.64
B	111.74±9.94	21.85±4.97
C	176.09±23.54	18.77±3.65
Statistic	F=82.37	F=3.93
	P=0.000	P=0.016

C and B Compared with Group-A, P<0.05.

## DISCUSSION

The Achilles tendon is the most robust tendon in the human body. The incidence of the epidemiological statistics of the rupture of the Achilles tendon is 0.18%, which is mainly the open cut and sports injury. After the repair, the rupture rate was about 24%. Therefore, surgical repair of Achilles tendon rupture is not easy. Although all kinds of sutures are applied to the clinic, the clinical effect is different up to now, there is no good suture to suture the rupture of the Achilles tendon.^3^ Considering whether a method is superior to other methods, it is necessary to first see that it is suturing the strength of the broken end of the tendon, and the second kind of suture has less interference with the Achilles tendon tissue. In order to find a good method of suture of Achilles tendon, it can promote the healing of the Achilles tendon and early rehabilitate. The scholars at home and abroad have done a lot of research on the suture of the Achilles tendon.^4^

Suture method has a crucial impact on the initial stability of Achilles tendon after suture. Currently, the widely used sutures include Kessler suture, Bunnell suture, and Bosworth suture. Watson^5^ confirmed that Bosworth suture can provide better biomechanical properties than Kessler suture and Bunnell suture, and it can meet the requirements of early functional exercise, Because Krackow suture can provide good biomechanical properties, it is often used as a control group to evaluate new suture methods in biomechanical research involving Achilles tendon suture.^6^-^9^ However, due to its own defects, Bosworth is not an ideal Achilles tendon suture. Strickland^10^ concluded that the ideal method of tendon suture must meet the following characteristics: simple operation, reliable fixation, adequate tensile strength, small gap formation, little disturbance to normal Achilles tendon, and little influence on blood supply.

In this experiment, the advantages of three stitching methods in tensile resistance were compared. When the instantaneous force (maximum fracture load) of the specimen is broken, the anchor method is higher than the other two groups. There is a difference in tension load between the anchor method and the Bunnell and Bosworth suture methods. This set of data the maximum load values were higher than those reported in the literature, the main reason is that in the experiment by using 1-0 absorbable Vicryl line, the line tension is 97.9N per share.

In this study, we compared the initial stability of three different suture methods after suture, and evaluated the immediate biomechanical properties of anchor suture to repair Achilles tendon laceration. Compared with Bunnel suture and Bosworth suture, stitching with wire anchors can provide worse immediate biomechanical properties. When the clinical suture is used to repair the rupture of Achilles tendon, the ability of calcaneus to resist tensile deformation is as important as the maximum load. Mullaney MJ et al.^11^ confirmed that the decrease of plantar flexor muscle strength after Achilles tendon repair is related to the excessive extension of Achilles tendon after operation.

Bosworth suture using gastrocnemius muscle aponeurosis is often used to treat closed Achilles tendon rupture. The curative effect is positive, a part of which may be related to the healing mechanism and stitching efficiency. The healing of the Achilles tendon includes endogenous and exogenous. Exogenous healing grows into the Achilles tendon by surrounding granulation tissue, and finally the scar is healed. This process is a combination of healing and adhesion. Endogenous healing is achieved by secreting collagen and rearranging it, which can effectively prevent adhesion. The healing of the rupture of the Achilles tendon is a unified process of endogenous healing and exogenous healing. In order to improve the curative effect, the proportion of exogenous healing must be reduced. Some scholars have proposed to use the flipping gastrocnemius muscle membrane to wrap the Achilles tendon, Applied Hong film mulching, which can effectively avoid the adhesion after the repair of the Achilles tendon.^12^

With anchor suture method, widely used in suture of flexor and extensor tendon and Achilles tendon, Generally, suitable for the cases with regular rupture of the Achilles tendon, the suture is strengthened around the ends of the broken ends, and the suture mechanism is to transform the tension of the line to the transverse pressure. The suture of the flexor tendon can provide strong enough tension, but the biomechanical strength of the Achilles tendon may be slightly different. Herbort^13^ is given a load test by the load assumed by the human Achilles tendon in human daily life, compared with one load and repeated cyclic loading. There is no difference in tensile strength between suture anchor suture method and Bunnell method after using same material suture. The maximum load mainly depends on the biomechanical property of suture material.

Achilles tendon is mainly longitudinal tendinous fibrous tissue. there is a small amount of tendon cells, elastic fibers and sugar polymer is the three largest component of the Achilles tendon is directly related to the effective way of nutrition, after the rupture of the Achilles tendon repair quality and its existing problems, loss plagued surgeon and tendon suture after biomechanical strength and limb function even sticky after the healing. The influence factors of tendon healing:

1. The early activities of control can promote tendon suture activities, long-term immobilization will make the proliferation of tendon cells decreased.
2. Tendon injury, stab, effect of stitching not valid for the broken end of the blood supply, affect the tendon itself, need to rely on the surrounding tissue repair the adhesion, increase the opportunity.

Therefore, suture should make the suture of the tendon able to bear greater tension as far as possible, and the suture material is exposed as little as possible, and not to split the tendon.^14^ The limitation of the Bunnell method and Kessler method for traditional Achilles tendon repair, splitting the pure Bunnell and Kessler suture method of tendon tension is too large may cause tendon. The suture anchor suture has the following advantages:

- Anchors fixed to the calcaneus, suture anchors in connection, reliable fixation, the distal end of Achilles tendon not because of Achilles tendon less split,
- No longer use steel wire suture, reduce the transfer operation of the treatment^15^, while reducing skin compression the chance of infection and wound.
